# Supplementary material for: Causal Relationship between Aging and Anorexia Nervosa: A White-Matter-Microstructure-Mediated Mendelian Randomization Analysis
Source: Biomedicines. 2024 Aug 16;12(8):1874. doi: 10.3390/biomedicines12081874 (PMC11351342; doi:10.3390/biomedicines12081874)
Supplement: Supplementary file 1 [file biomedicines-12-01874-s001.zip › Supplementary Figure/Figure S9/Leaveoneout plot of MR analysis for ubm-b-1899 to AN.pdf]

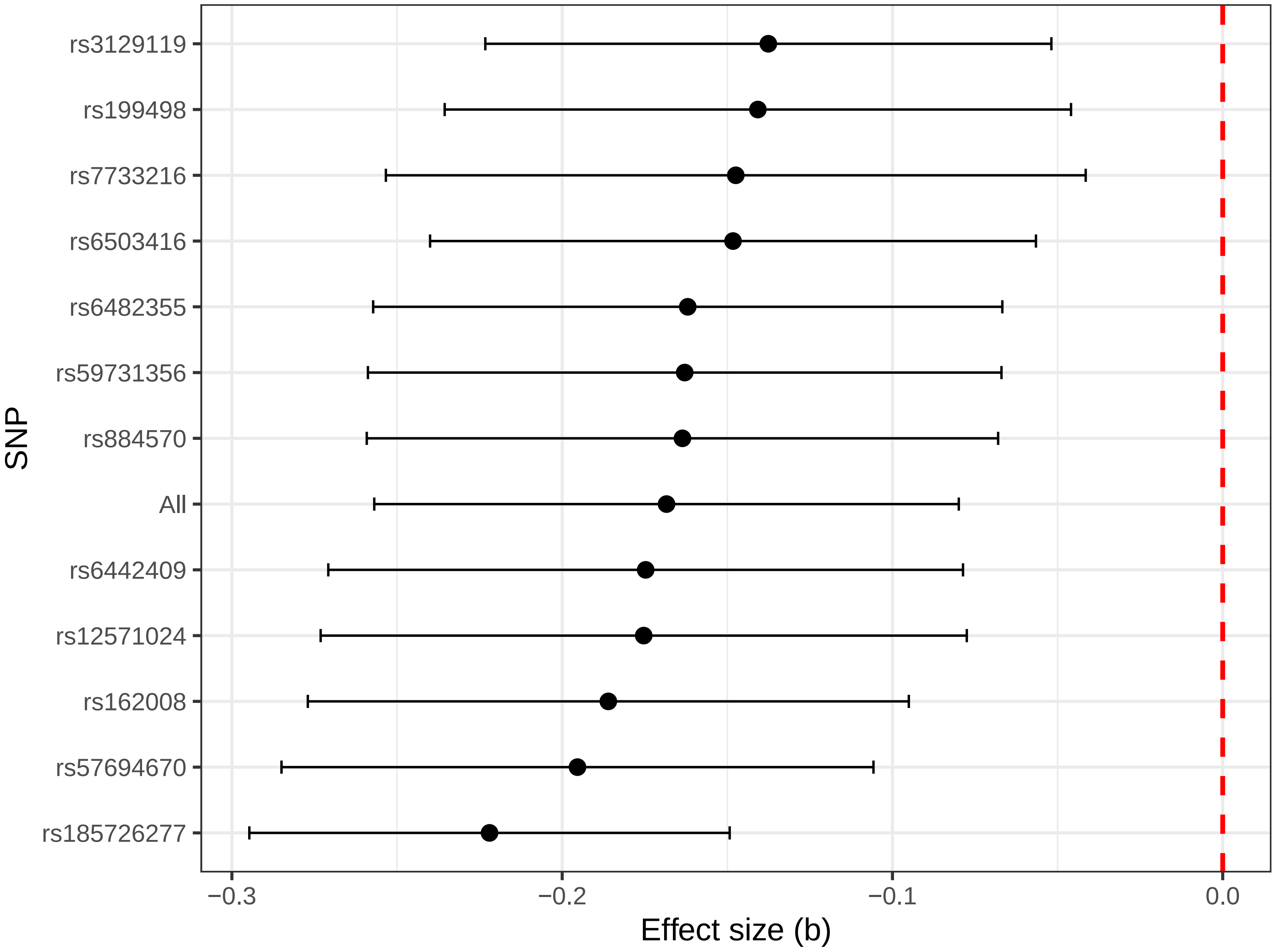

rs3129119
rs199498
rs7733216
rs6503416
rs6482355
rs59731356
rs884570
All
rs6442409
rs12571024
rs162008
rs57694670
rs185726277
SNP
−0.3
−0.2
−0.1
0.0
Effect size (b)
